# Consensus-Guided Construction of H5N1-Specific and Universal Influenza a Multiepitope Vaccines

**DOI:** 10.3390/biology14101327

**Published:** 2025-09-25

**Authors:** Marco Palma

**Affiliations:** 1 Institute for Globally Distributed Open Research and Education (IGDORE), 03181 Torrevieja, Spain; marco.palma@igdore.org; 2 Biointelix LLC, Sheridan, WY 82801, USA

**Keywords:** influenza A, H5N1, universal vaccine, multiepitope design, hemagglutinin receptor-binding domain, immunoinformatics, toll-like receptors, molecular docking, immune simulation, cross-protective immunity

## Abstract

Background/Objectives: Influenza A viruses—including highly pathogenic H5N1—remain a global threat due to rapid evolution, zoonoses, and pandemic potential. Strain-specific vaccines targeting variable antigens often yield limited, short-lived immunity. The HA receptor-binding domain (RBD), a functionally constrained and immunologically relevant region, is a promising target for broad and subtype-focused vaccines. We aimed to design multiepitope constructs targeting conserved HA-RBD and adjacent domains to elicit robust, durable, cross-protective responses. Methods: Extensive sequence analyses (>20,000 H5N1 and >190,000 influenza A sequences) were used to derive consensus sequences. Three HA-based candidates were developed: (i) EpitoCore-HA-VX, a multi-epitope construct containing CTL, HTL, and B-cell epitopes from the H5N1 HA-RBD; (ii) StructiRBD-HA-VX, incorporating a conformationally preserved RBD segment; and (iii) FusiCon-HA-VX, targeting the conserved HA fusion peptide shared across subtypes. Two external HA comparators—a 400-aa HA fragment and the literature-reported HA-13–263-Fd-His—were analyzed under the same pipeline. The workflow predicted epitopes; evaluated antigenicity, allergenicity, toxicity, conservation, and HLA coverage; generated AlphaFold models; performed TLR2/TLR4 docking with pyDockWEB; and carried out interface analysis with PDBsum; and C-ImmSim simulations. Results: Models suggested stable, energetically favorable TLR2/TLR4 interfaces supported by substantial binding surfaces and complementary electrostatic/desolvation profiles. Distinct docking patterns indicated receptor-binding flexibility. Immune simulations predicted strong humoral responses with modeled memory formation and, for the H5N1-focused designs, cytotoxic T-cell activity. All candidates and comparators were predicted to be antigenic, non-allergenic, and non-toxic, with combined HLA coverage approaching global breadth. Conclusions: This study compares three design strategies within a harmonized framework—epitope collation, structure-preserved RBD, and fusion-peptide targeting—while benchmarking against two HA comparators. EpitoCore-HA-VX and StructiRBD-HA-VX showed promise against diverse H5N1 isolates, whereas FusiCon-HA-VX supported cross-subtype coverage. As these findings are model-based, they should be interpreted qualitatively; nonetheless, the integrated, structure-guided approach provides an adaptable path for advancing targeted H5N1 and broader influenza A vaccine concepts.

## Simple Summary

Influenza A viruses, such as the bird flu strain H5N1, remain a serious threat to global health because they change rapidly, can spread from animals to humans, and have the potential to cause pandemics. Current vaccines often focus on parts of the virus that mutate frequently, which limits their effectiveness and leads to only short-term protection. In this study, we used computer-based methods to design and compare three new vaccine candidates that target more stable regions of the influenza virus. One design focused on combining many small immune-triggering fragments, another preserved the natural shape of a critical binding region, and the third targeted a highly conserved fusion element that is shared by different influenza subtypes. Our analyses showed that these vaccine candidates are predicted to be safe, effective at stimulating both antibody and T-cell responses, and broadly protective across diverse virus strains. While these findings are based on modeling and require experimental validation, the study demonstrates how advanced computational tools can guide the development of next-generation influenza vaccines. This approach could help create longer-lasting, more universal protection and contribute to better preparedness against future influenza outbreaks.

## 1. Introduction

Influenza A viruses remain a significant global public health threat due to their high mutation rates, zoonotic transmission potential, and capacity to trigger pandemics [1]. Among them, highly pathogenic avian influenza (HPAI) H5N1 is of particular concern because of its exceptionally high human case fatality rate, sporadic yet severe outbreaks, and the ongoing risk of genetic reassortment that could facilitate efficient human-to-human transmission [2]. The continued evolution not only of H5N1 but also of other influenza A subtypes highlights the limitations of current vaccines, which are predominantly strain-specific and require frequent reformulation to match circulating variants. This challenge underscores the need for next-generation vaccines capable of eliciting broad, long-lasting protection against diverse influenza A viruses.

Hemagglutinin (HA) is the major surface glycoprotein of influenza A viruses and plays a critical role in viral entry [3]. It mediates the initial binding of the virus to sialic acid receptors on host epithelial cells and facilitates membrane fusion following endocytosis. Structurally, HA consists of a highly variable globular head domain and a more conserved stem (or stalk) domain [4]. While the head domain contains the receptor-binding domain (RBD) and is a dominant target for strain-specific neutralizing antibodies, the stem domain harbors conserved epitopes shared across multiple influenza subtypes.

In the case of H5N1, HA is the primary determinant of host range, transmissibility, and antigenicity, making it an ideal antigen for vaccine design [5]. Targeting HA can induce neutralizing antibodies that block viral attachment and fusion [6], effectively preventing infection. Importantly, the conserved nature of the HA stem makes it a promising focus for universal influenza vaccine strategies. Vaccines designed to elicit broadly neutralizing antibodies against these conserved stem epitopes could provide cross-protection not only against H5N1 but also against a wide range of influenza A subtypes, reducing the need for frequent vaccine updates and enhancing pandemic preparedness.

Recent advances in computational vaccinology and epitope-based vaccine design offer promising alternatives [7]. By focusing on conserved and functionally relevant regions such as the HA-RBD, multiepitope vaccines can be engineered to stimulate robust humoral and cellular immune responses. These constructs can incorporate cytotoxic T lymphocyte (CTL), helper T lymphocyte (HTL) [8], and B-cell epitopes [9], optimized for antigenicity, population coverage, and low allergenicity using immunoinformatics tools.

Despite their potential, many previous vaccine designs have primarily focused on B-cell epitopes [10,11] or have overlooked key factors such as structural integrity and T-cell epitope diversity. In particular, the preservation of epitope conformation—especially in structure-dependent regions such as the HA-RBD—remains an area that could benefit from further exploration.

In this study, three multi-epitope vaccine constructs were designed targeting the HA-RBD and adjacent regions, including two constructs specifically for H5N1 and another incorporating highly conserved segments across influenza A subtypes for a universal vaccine approach. The design strategy incorporated both linear and conformational epitopes, ensuring structural fidelity in regions critical for immune recognition. Structural modeling, molecular docking, and immune simulations were employed to assess the predicted interactions of these constructs with Toll-like receptors and to evaluate their potential to elicit robust, non-allergenic, and non-toxic immune responses. This work presents a bioinformatics-driven, laboratory-free framework for rational vaccine design, serving as a critical preclinical step toward the development of broadly protective influenza A vaccines.

## 2. Materials and Methods

### 2.1. Sequence Retrieval

HA protein sequences from various H5N1 influenza A virus isolates, as well as from other Influenza A subtypes of relevance to human and animal health, were retrieved from the Influenza Research Database (NCBI Virus) [12]. Full-length HA sequences were retrieved in FASTA format for subsequent bioinformatics analyses. Sequences were obtained using the “Advanced Filter for GenBank Sequences,” specifying Influenza A virus (taxid: 11320) and subtype-specific hemagglutinin entries, with sequence length restricted to 540–580 amino acids to ensure completeness. No restrictions on species, geographic origin, or collection date were applied, thereby capturing broad sequence diversity. The collected sequences were aligned with MAFFT v7 [13], and consensus sequences were generated in Jalview [14] by selecting the most frequent residue at each alignment position.

The amino acid sequence of the protein used as adjuvant in the vaccine construction was retrieved from the UniProt database [15]. Specifically, the 50 s ribosomal L7/L12 (UniProt ID: P9WHE3) from *Mycobacterium tuberculosis* (strain ATCC 25618/H37Rv) was selected. The full-length sequence was downloaded in FASTA format for subsequent analysis.

### 2.2. Frequency Analysis of Residues in the HA RBD for Vaccine Targeting

The retrieved HA sequences were categorized into two groups: the first included only H5N1 Influenza A virus isolates, aimed at designing a subtype-specific vaccine based on a consensus sequence derived from these isolates. The second group included HA sequences from multiple Influenza A subtypes, aiming to develop a universal vaccine by identifying conserved regions across subtypes. Consensus sequences from each subtype were aligned and compared to pinpoint the most conserved regions within or near the RBD of HA.

Multiple sequence alignment was performed using the MAFFT tool [16,17] (https://mafft.cbrc.jp/alignment/server/large.html, accessed on 20 September 2025), allowing for the generation of consensus sequences and the calculation of amino acid frequency within epitope regions or HA segments included in the RBD-based vaccine constructs. Alignment results were visualized using the Jalview software [14] version: 2.11.5.0 (https://www.jalview.org/, accessed on 20 September 2025).

### 2.3. Epitope Prediction

B-cell, cytotoxic T lymphocyte (CTL), and helper T lymphocyte (HTL) epitopes were predicted from the H5N1 hemagglutinin consensus sequence within residues 124 to 277, as defined by the reference isolate A/goose/Guangdong/1/1996 (H5N1), to guide the design of a subtype-specific H5N1 HA vaccine.

#### 2.3.1. B-Cell Epitope Prediction

The identification of B-cell epitopes within the RBD of the HA protein was performed using two complementary bioinformatics tools to predict both linear and conformational epitopes. Linear (continuous) B-cell epitopes were predicted using the ABCpred server [18] (https://webs.iiitd.edu.in/raghava/abcpred/ABC_submission.html, accessed on 20 September 2025). The analysis was conducted with a fixed window length of 16 amino acids and a threshold score of 0.51. Predicted epitopes with scores exceeding this threshold were considered significant and selected for subsequent analysis.

For the prediction of discontinuous (conformational) B-cell epitopes, the DiscoTope 3.0 server (https://services.healthtech.dtu.dk/services/DiscoTope-3.0/, accessed on 20 September 2025) was utilized [19]. As input for this tool, a three-dimensional structural model of the HA RBD was generated using the AlphaFold protein structure prediction platform. The resulting model was used to facilitate spatial epitope mapping via DiscoTope 3.0.

#### 2.3.2. CTL Epitope Prediction

Prediction of CTL epitopes was performed using the NetCTL 1.2 server [20] (https://services.healthtech.dtu.dk/services/NetCTL-1.2/, accessed on 20 September 2025) which integrates three key components of antigen processing: proteasomal cleavage, transporter associated with antigen processing (TAP) transport efficiency, and major histocompatibility complex (MHC) class I binding affinity. This comprehensive approach enables the identification of peptides with high potential to be presented on MHC class I molecules and recognized by CD8^+^ T cells.

For this analysis, three representative HLA class I supertypes—HLA-A2, HLA-A3, and HLA-B7—were selected based on their high prevalence and broad population coverage. These supertypes collectively provide estimated global population coverage of approximately 80–90%, making them particularly relevant for the development of broadly protective, epitope-based vaccine candidates.

#### 2.3.3. HTL Epitope Prediction

To identify potential HTL epitopes within the RBD, the IEDB MHC Class II binding prediction tool was utilized. Epitope prediction was performed using the NetMHCIIpan 4.1 EL method [21] (http://tools.iedb.org/mhcii/, accessed on 20 September 2025) with default parameters. The analysis was restricted to the human host, specifically targeting the HLA-DR locus due to its immunodominant role in antigen presentation.

A comprehensive reference panel of HLA-DR alleles—representing approximately 99% of the global population—was employed to ensure broad population coverage. Predicted epitopes were ranked based on their percentile scores, with lower percentile ranks corresponding to higher predicted binding affinities. Epitopes with the strongest binding predictions were selected for subsequent analysis.

### 2.4. Identification of IFN-γ–Inducing Epitopes

Interferon-gamma (IFN-γ) plays a central role in regulating and enhancing cellular immune responses, making it a critical marker for evaluating vaccine efficacy. To identify epitopes with the potential to induce IFN-γ production, the previously selected HTL epitopes were analyzed using the IFNepitope prediction server [22] (http://crdd.osdd.net/raghava/ifnepitope/predict.php, accessed on 20 September 2025)

The analysis employed a hybrid prediction model that integrates motif-based recognition with a Support Vector Machine (SVM) learning algorithm. This approach enables accurate discrimination between IFN-γ–inducing epitopes and those associated with alternative cytokine responses. Epitopes predicted to be strong IFN-γ inducers were prioritized for further immunological evaluation. In the Model for prediction option was selected IFN-gamma versus other cytokine.

### 2.5. Toxicity Prediction

The selection of non-toxic epitopes is essential for ensuring the safety and tolerability of subunit vaccine candidates. To assess the toxicity of the predicted epitopes, the ToxinPred server [23] was employed. Only epitopes classified as non-toxic were retained for inclusion in the final vaccine construct.

In parallel, allergenicity assessment was conducted using AllerCatPro 2.0 [24] (https://allercatpro.bii.a-star.edu.sg/, accessed on 20 September 2025). This multi-tool approach provided a comprehensive evaluation of potential allergenic risks, enhancing the safety profile of the designed vaccine.

### 2.6. Vaccine Design

The vaccine design process initially focused on enhancing an existing adjuvant for use in the formulations. Subsequently, three distinct vaccine constructs were designed.

#### 2.6.1. Adjuvant Development

An adapted form of the 50S ribosomal protein L7/L12 from *M. tuberculosis* (UniProt ID: P9WHE3), previously reported as a promising vaccine adjuvant [25], was employed in this study. A truncated fragment encompassing amino acid residues 65 to 130 was selected based on its reported enhancement of immunogenic properties while preserving the native tertiary structure of the parent protein.

#### 2.6.2. Epitope-Based Vaccine Construct Targeting the RBD of H5N1 Hemagglutinin

The first approach involved selecting the top three epitopes from each category—linear B-cell, conformational B-cell, CTL, and HTL epitopes—and linking them sequentially along with a truncated 50S ribosomal protein L7/L12 adjuvant. This construction primarily focused on linear epitopes and did not prioritize preserving the native tertiary structure of the RBD. The epitopes were connected using specific linkers to optimize immunogenicity and structural flexibility: linear B-cell epitopes were linked by KK linkers, CTL epitopes by AAY linkers, and HTL epitopes by GPGPG linkers. The adjuvant was fused at the N-terminus of the epitope assembly via an EAAAK. The resulting vaccine construct was named EpitoCore-HA-VX.

#### 2.6.3. Structural Segment-Based Vaccine Derived from the H5N1 HA RBD

The second design strategy centered on preserving the native conformation of the RBD by selecting a segment that included at least one of the three predicted conformational B-cell epitopes. The sequence corresponds to residues 188 to 255 of hemagglutinin (HA), as defined by the reference isolate A/goose/Guangdong/1/1996 (H5N1) (Proteome ID: UP000131152; UniProt ID: Q9Q0U6) (https://www.uniprot.org/uniprotkb/Q9Q0U6/entry, accessed on 20 September 2025). In this case, the truncated L7/L12 adjuvant was similarly attached at the N-terminus using the EAAAK linker, ensuring the structural integrity of the RBD fragment was maintained. This vaccine construct was designated StructiRBD-HA-VX.

These two complementary vaccine constructs allowed for comparative evaluation of a primarily linear epitope-based vaccine versus one emphasizing conformational epitope preservation within the RBD structure.

#### 2.6.4. Universal Influenza a Vaccine

A conserved region (RGLFGAIAGFIEGGWQGMVDGWYG), spanning residues 346–369 of the HA sequence from the reference isolate A/goose/Guangdong/1/1996 (H5N1), was fused to the C-terminal end of the truncated L7/L12 adjuvant via an EAAAK linker. The resulting construct was designated FusiCon-HA-VX.

#### 2.6.5. Full-Length HA Fragment Control

In addition to the three vaccine constructs, a 400 amino acid fragment corresponding to residues 1–400 of the consensus hemagglutinin (HA) sequence of H5N1 was included as a control to benchmark the design workflow. The consensus sequence was generated from multiple H5N1 HA isolates to capture the most representative form of the protein.

This HA fragment was subjected to the same computational pipeline applied to the designed vaccines. The tertiary structure was predicted using AlphaFold 3 (https://alphafoldserver.com/, accessed on 20 September 2025), followed by structural refinement with the GalaxyRefine server (https://galaxy.seoklab.org/cgi-bin/submit.cgi?type=REFINE, accessed on 20 September 2025). Structural quality was assessed through ProSA-web (https://prosa.services.came.sbg.ac.at/prosa.php, accessed on 20 September 2025), ERRAT, and PROCHECK analyses (https://saves.mbi.ucla.edu/, accessed on 20 September 2025) to confirm stereochemical plausibility and overall reliability. The refined structure was subsequently used for protein–protein docking with the host receptor sialic acid-binding domain via pyDockWEB (https://life.bsc.es/pid/pydockweb, accessed on 20 September 2025). Docking results were visualized and analyzed with PDBsum1 (https://www.ebi.ac.uk/thornton-srv/databases/pdbsum/Generate.html, accessed on 20 September 2025) to characterize key interface residues and hydrogen-bonding patterns.

Finally, the immunogenic potential of the HA fragment was evaluated using C-ImmSim (https://kraken.iac.rm.cnr.it/C-IMMSIM/index.php?page=0, accessed on 20 September 2025) to simulate humoral and cellular immune responses. The simulation outputs included antibody isotype dynamics, cytokine levels, and T-cell activation patterns. This control served as a reference to contextualize the immune and structural profiles of the three engineered vaccine constructs.

### 2.7. Structural Modeling, Refinement, and Structural Quality Assessment

The three-dimensional structures of the HA-RBD and the designed vaccine constructs were predicted using AlphaFold [26]. For each construct, five models were generated and subsequently refined using GalaxyRefine [27], which enhances atomic-level accuracy through iterative perturbation and molecular dynamics-based relaxation. This process yielded a total of 25 refined models.

Structural quality assessment was performed using PROCHECK [28], focusing on key quality indicators derived from Ramachandran plot statistics, including the percentage of residues in favored, allowed, generously allowed, and disallowed regions. Additional quality metrics included G-factor scores (reflecting stereochemical plausibility), the number of steric clashes (bad contacts), and deviations in bond lengths, bond angles, and residue geometries, including side-chain orientations.

This comprehensive assessment enabled the identification of the most structurally reliable models from each prediction, which were selected as top candidates for subsequent molecular docking studies. Finally, all selected refined models were visualized and examined using UCSF ChimeraX 1.10 [29] (https://www.rbvi.ucsf.edu/chimerax/, accessed on 20 September 2025), facilitating detailed inspection of their spatial conformations and molecular features.

### 2.8. Molecular Docking Analysis of Vaccine–TLR Interactions

To evaluate the potential binding interactions between the vaccine constructs and innate immune receptors, molecular docking simulations were conducted. The docking procedure was carried out using the pyDOCKweb server [30], which applies a rigid-body docking algorithm optimized for predicting protein–protein interactions. The receptor molecules used for docking were Toll-like receptor 2 (TLR2) (PDB ID: 6NIG) and Toll-like receptor 4 (TLR4) (PDB ID: 4G8A).

Docking results were assessed based on global binding energy scores, and the complex with the lowest predicted binding energy was selected for further analysis of intermolecular interactions. ChimeraX was used to visualize the docked complexes and examine the spatial arrangement and orientation of the vaccine at the receptor interface.

To identify and characterize specific inter-residue contacts within the docked complexes, the PDBsum web server was employed. This tool provides detailed interaction maps, including hydrogen bonds, salt bridges, and hydrophobic contacts, thereby offering insights into the binding interfaces between the vaccine constructs and TLR receptors.

### 2.9. In Silico Simulation of Host Immune Response

To evaluate the immunogenic potential and dynamics of host immune activation in response to the designed vaccine, an in silico immune simulation was performed using the C-ImmSim server [31]. This agent-based simulation platform models the mammalian immune system, incorporating key components of both innate and adaptive immunity, and is capable of predicting immune response profiles based on position-specific scoring matrices (PSSMs), machine learning algorithms, and empirical immunological principles.

The simulation was configured to mimic a typical human vaccination schedule involving three doses administered at four-week intervals, which correspond to simulation time steps 1, 85, and 169. These intervals are designed to reflect primary exposure followed by booster immunizations, enabling the assessment of both primary and memory immune responses.

The simulation environment was set to a volume parameter of 50 (arbitrary units), with a total of 1000 time steps to capture both the acute and long-term immunological events post-vaccination. For each injection, 2000 antigen units were administered, and an adjuvant strength of 100 was used to enhance immune activation. The simulation was conducted under a lipopolysaccharide (LPS)-free condition, which allows for the specific evaluation of vaccine-induced responses without nonspecific endotoxin effects. A fixed random seed value of 12345 was used to ensure reproducibility and consistency across multiple simulation runs.

C-ImmSim output data included quantitative measurements and graphical representations of immune parameters such as B-cell and T-cell population dynamics, antibody titers (IgM, IgG1, IgG2, IgA, and IgE), cytokine profiles, and the formation of memory cells, providing a comprehensive overview of the expected immunological performance of the vaccine construct.

### 2.10. Population Coverage Estimation

Given the extensive polymorphism in human leukocyte antigen (HLA) alleles across global populations, assessing the potential population coverage of the designed multi-epitope vaccine is critical. To evaluate the theoretical distribution and immunological reach of the selected epitopes, we utilized the Population Coverage tool provided by the Immune Epitope Database (IEDB) [32]. This tool estimates the proportion of individuals—across different geographical regions—likely to present at least one epitope based on their HLA genotypes.

The analysis was performed using default parameters, incorporating the HLA restriction data for all selected epitopes. Both HLA class I and class II alleles were included in the assessment. Specifically, emphasis was placed on prevalent HLA class I supertypes (HLA-A2, HLA-A3, and HLA-B7) and class II alleles (HLA-DR). This approach allowed us to predict the global and regional population segments that may mount an immune response to the vaccine construct.

### 2.11. Codon Adaptation and In Silico Cloning

To enhance the potential expression of the designed vaccine construct and the 400aa Ha fragment in a bacterial host system, the amino acid sequence was reverse-translated and optimized for *Escherichia coli* K12 codon usage. This was performed using the Java Codon Adaptation Tool (JCat) [33], which calculates codon adaptation index (CAI) and GC content to assess translational efficiency. Restriction enzyme recognition sites for *Xho*I and *BamH*I were appended to the 5′ and 3′ ends of the sequence, respectively, to facilitate downstream cloning. The codon-optimized sequence was then inserted into the pET28a(+) expression vector using the SnapGene 8.2 software (GSL Biotech, Boston, MA, USA) for visualization and verification of the cloning strategy

## 3. Results

### 3.1. Protein Sequence Retrieval

The 130-amino acid sequence of the 50S ribosomal protein L7/L12 was retrieved from the UniProt database for use as an adjuvant. The truncated 50S ribosomal protein L7/L12 used in this study had the following sequence VILEAAGDKKIGVIKVVREIVSGLGLKEAKDLVDGAPKPLLEKVAKEAADEAKAKLEAAGATVTVK.

A large dataset of HA sequences from various Influenza A subtypes was retrieved from the Influenza Research Database (NCBI Virus) (https://www.ncbi.nlm.nih.gov/labs/virus/vssi/#/, accessed on 20 September 2025) (Table 1), including over 20,000 HA sequences from diverse H5N1 isolates.

### 3.2. Comparative Analysis of Residue Conservation in Influenza a Subtypes

#### 3.2.1. Consensus HA Sequence Among H5N1 Isolates

**Figure 1:**
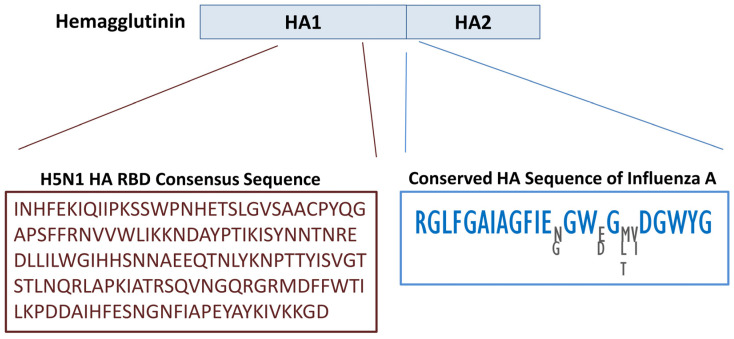
Upper panel: Structural representation of the two main domains of hemagglutinin (HA). Lower left panel: Consensus HA RBD derived from H5N1 isolates. Lower right panel: Conserved region spanning the HA1–HA2 cleavage site and the N-terminal fusion peptide of HA2. While most residues are invariant across multiple Influenza A subtypes, four positions show limited variability, and the alternative residues at these sites are displayed.

A consensus HA sequence was derived from the analysis of over 20,000 HA sequences from diverse H5N1 isolates, representing the most frequent amino acid at each position. The segment spanning residues 124–277 within the HA1 domain was selected as the reference (master) sequence for subsequent analyses of the HA RBD (Figure 1, lower left panel).

#### 3.2.2. Conservation of the HA Sequence Across Influenza a Subtypes

HA sequences from different Influenza A subtypes were analyzed, with particular focus on the RBD and adjacent regions. Consensus sequences for each subtype of interest were first generated through multiple sequence alignment. These consensus HA sequences were then aligned with each other to identify conserved regions shared among subtypes.

Within the HA1 region, no continuous stretches of identical residues were detected, although some individual residues were conserved. In contrast, a highly conserved segment was identified starting from the last strongly conserved residue in HA1 and extending 23 residues into the N-terminal region of HA2. This segment contained 20 highly conserved residues (Figure 1, lower right panel) and corresponds to the fusion peptide, a critical element that mediates viral–host membrane fusion and is essential for viral entry.

### 3.3. Epitopes Prediction

B-cell epitopes are critical components of the adaptive immune response, as they are recognized by B-cell receptors, leading to the production of immunoglobulins. Identifying these epitopes is essential for designing vaccines that accurately mimic the immune response induced by natural infection and promote long-lasting immunity. Linear B-cell epitopes, each 16 amino acids in length, were predicted from the HA RBD sequence using the ABCpred server with a threshold score of 0.51. The identified epitopes are presented in Table 2. The top three epitope with scores of 0.94, 0.94, and 0.89 was chosen to be included in a multiple epitope vaccine.

In addition, to identify conformational B-cell epitopes, it was necessary to predict the three-dimensional structure of the receptor-binding domain of hemagglutinin. This was accomplished using AlphaFold. The resulting model achieved a predicted Template Modeling score (pTM) of 0.91, indicating a highly reliable global structure prediction. This suggests that the overall fold and domain organization are accurate and suitable for downstream structural and functional analyses.

**Figure 2:**
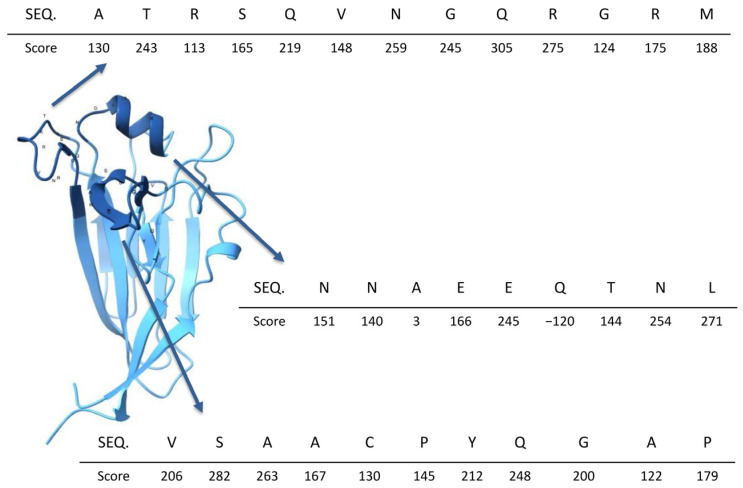
Location of the primary conformational B-cell epitopes predicted in the RBD. The figure displays the amino acid sequences of the corresponding epitopes, with the score for each position shown directly beneath the sequences.

Conformational epitopes were identified using DiscoTope 3.0, which assigns a score to each residue in the protein structure based on its likelihood of being part of a conformational epitope. Residues with scores above 100 were selected, as such values indicate a high probability of being part of an epitope. These residues highlight regions forming a surface-accessible pocket, which is likely involved in binding to sialic acid-containing receptors on the host cell surface. The predicted structural model and the mapped conformational epitopes are illustrated in Figure 2.

CTL epitopes are short peptides (typically 9 amino acids in length) presented by MHC class I molecules on the surface of infected or abnormal cells. These epitopes are recognized by CD8^+^ T cells, which subsequently eliminate the presenting cells. This immune mechanism is essential for clearing virus-infected cells, eliminating cancerous or transformed cells, and controlling intracellular pathogens.

In this study, ten 9-mer CTL epitopes were predicted and ranked based on their binding scores and association with HLA class I supertypes (Table 3). The highest-ranking epitopes corresponding to the HLA-A2, HLA-A3, and HLA-B7 supertypes were selected for further analysis, with one representative epitope chosen for each supertype.

HTL epitopes, presented by MHC class II molecules, are crucial in vaccine design because they activate CD4^+^ T cells, which orchestrate and sustain immune responses. HTLs enhance B cell activation and antibody production, support CTL responses, and promote immunological memory. Including HTL epitopes in vaccines ensures a broader, longer lasting, and more balanced immune response, especially against complex pathogens or cancer.

Ten HTL epitopes were predicted (Table 4) and ranked based on their percentile scores, which reflect the relative binding affinity of each peptide to MHC class II molecules compared to a reference peptide set. A lower percentile rank indicates stronger predicted binding affinity. Subsequent analyses confirmed that all predicted epitopes are non-allergenic, and capable of inducing IFN-γ responses.

### 3.4. Vaccine Construction

The vaccine construct was designed to begin with a truncated fragment of the 50S ribosomal protein, specifically comprising amino acid residues 65–130, which serves as a molecular adjuvant. Antigenicity prediction analysis yielded a score of 0.4583 for the truncated variant, surpassing the model’s threshold of 0.4 for classification as antigenic. In contrast, the full-length protein scored 0.382 and was classified as non-antigenic. These results support the selection of the truncated form as a promising candidate for use as an adjuvant based on its structural integrity and improved antigenicity.

**Figure 3:**
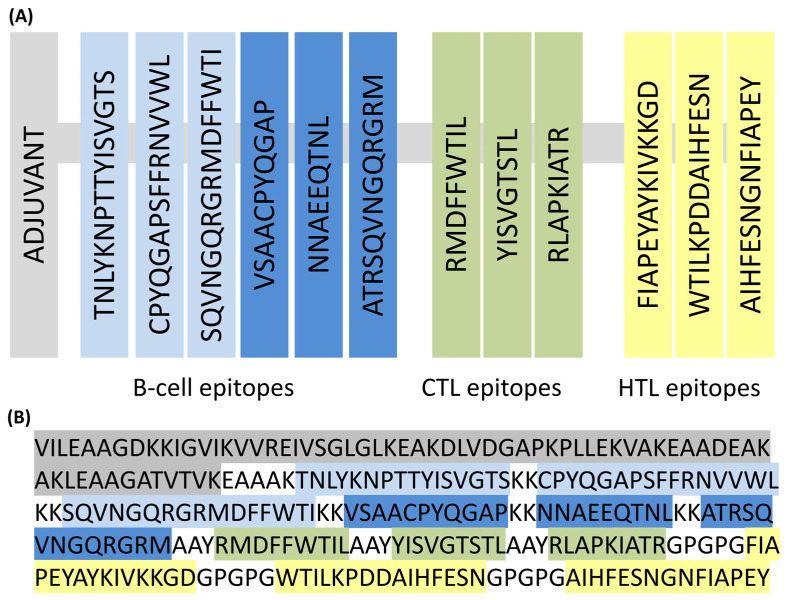
Design of the multi-epitope HA-RBD vaccine EpitoCore-HA-VX. (**A**) Schematic illustration of the overall organization of the vaccine construct, highlighting the adjuvant and epitope arrangement. (**B**) Primary amino acid sequence of the construct, beginning with a truncated 50S ribosomal protein segment (adjuvant, shown in grey) fused at the N-terminus via an EAAAK linker. This is followed by linear B-cell epitopes (light blue), conformational B-cell epitopes (dark blue), cytotoxic T lymphocyte (CTL) epitopes (green), and helper T lymphocyte (HTL) epitopes (yellow). B-cell, CTL, and HTL epitopes are interconnected using KK, AAY, and GPGPG linkers, respectively.

Following the adjuvant region, the construction includes a series of HA-RBD epitopes arranged in tandem. These epitopes have been strategically selected for their immunological relevance and are connected via short, flexible linkers to ensure optimal spatial orientation and epitope presentation. The detailed organization of the HA-RBD epitopes, including their amino acid sequences and the linker regions used to join them, is illustrated in Figure 3. This vaccine construct was designated as EpitoCore-HA-VX.

**Figure 4:**
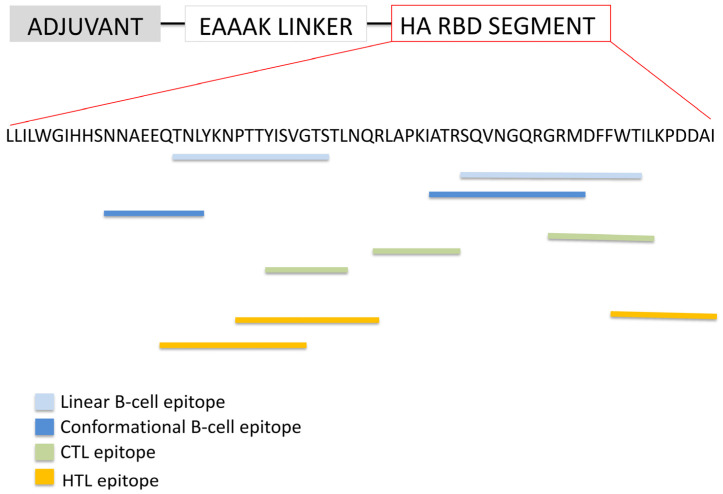
Diagram illustrating the structural organization of the StructiRBD-HA-VX vaccine construct. The upper panel illustrates the organization of the different components of the vaccine construct, including the adjuvant and epitope regions. The lower panel presents the amino acid sequence of the selected HA-RBD segment, highlighting the predicted epitopes within this region. Both linear and conformational B-cell epitopes, as well as CTL and HTL epitopes, are indicated to demonstrate the immunogenic potential of the construct.

The second vaccine construct features a truncated segment of the 50S ribosomal protein at the N-terminus, linked via an EAAAK linker to a defined region of the HA RBD, encompassing residues 188 to 255 (Figure 4). Structural prediction analyses indicate that this RBD fragment retains its native three-dimensional conformation, thereby supporting the preservation of epitope integrity and enhancing the likelihood of eliciting a robust immune response. This segment includes at least two predicted linear B-cell epitopes, two conformational B-cell epitopes, and three CTL epitopes, underscoring its immunological relevance. This vaccine construct was designated as StructiRBD-HA-VX.

**Figure 5:**
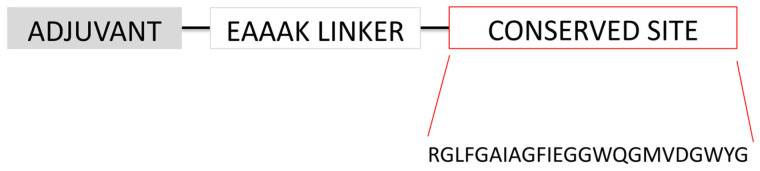
Schematic representation of the FusiCon-HA-VX vaccine construct and its structural organization.

The third vaccine construct incorporates a highly conserved 24-residue segment (Figure 5), identified through multiple sequence alignment of nearly 20,000 hemagglutinin sequences from diverse influenza A subtypes. This consensus sequence is located at the N-terminal region of the HA2 subunit, marking the beginning of the ectodomain and lying adjacent to the HA1 subunit. Notably, this segment encompasses the fusion peptide, a critical element required for mediating viral entry by facilitating membrane fusion between the virus and the host cell.

### 3.5. Toxicity Prediction and Allerginicity

Allergenicity analysis using AllerCatPro 2.0 indicated that all three vaccine constructs are unlikely to trigger allergic responses. The tool classified the constructs as non-allergens based on an integrated assessment of amino acid sequence similarity, structural features, and physicochemical properties compared to known allergens.

### 3.6. Structural Prediction

The structure of the RBD of hemagglutinin was predicted using AlphaFold, yielding a model with a predicted Template Modeling score (pTM) of 0.91. This high pTM value indicates a highly reliable global structure prediction, suggesting that the overall fold and domain arrangement are accurate and suitable for downstream structural or functional analyses.

**Figure 6:**
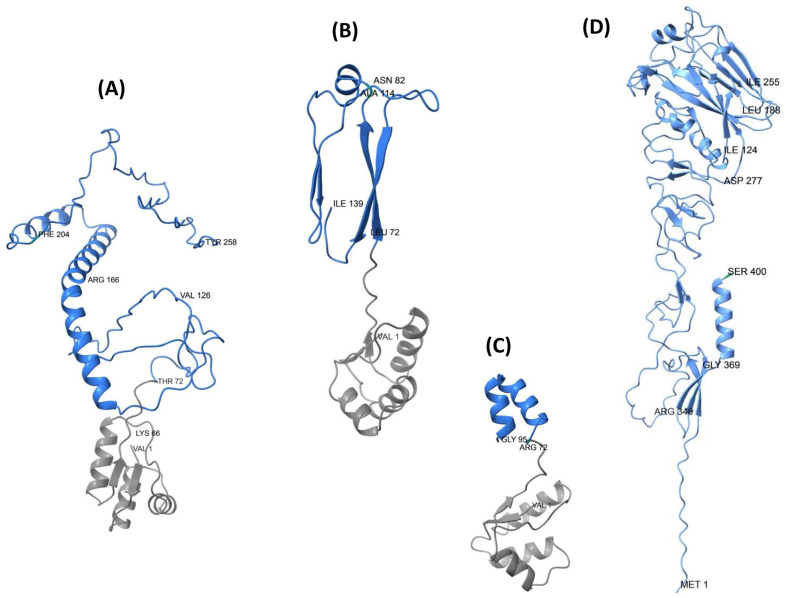
Predicted Three-Dimensional Structures of the Designed HA-RBD Vaccine Constructs. Ribbon representations of the predicted three-dimensional structures for the vaccine candidates EpitoCore-HA-VX (**A**), StructiRBD-HA-VX (**B**), and FusiCon-HA-VX (**C**) generated using AlphaFold. Panel (**D**) shows the consensus HA fragment control encompassing residues 1–400 of H5N1 HA. The blue region represents the RBD or their epitopes, while the grey region corresponds to the adjuvant component incorporated into the vaccine constructs. For each structure, the first N-terminal residue and the last C-terminal residue are shown, along with selected key residues that highlight important functional regions.

Structural predictions of the three designed HA-RBD vaccine constructs revealed notable differences in their conformational organization (Figure 6A–C). The EpitoCore-HA-VX construct exhibited a more disordered conformation within the epitope-rich regions, characterized by the presence of some α-helices and a high proportion of random coils, deviating notably from the native structural features of the HA RBD in the corresponding region.

In contrast, the StructiRBD-HA-VX construct displayed a more ordered and stable conformation, also composed of α-helices and β-sheets, but more closely resembling the native HA-RBD architecture.

The FusiCon-HA-VX vaccine, designed based on a conserved segment, contains two short α-helices located within the HA region of the vaccine.

### 3.7. Structural Refinement and Structural Quality Assessment

AlphaFold generated five structural models for every HA RBD vaccine candidate, resulting in a total of 15 initial models. These models were subsequently refined using GalaxyRefine to enhance their structural accuracy and quality, resulting in five structural refinement per AlphaFold model. Structural quality assessment of each refined model was performed using PROCHECK to evaluate stereochemical quality and geometric parameters across models.

PROCHECK analysis of all GalaxyRefine-processed models identified model 2.5 of the EpitoCore-HA-VX construct as the most suitable candidate for docking studies, based on its superior structural quality. This model demonstrated the highest G-factor value (0.21), reflecting excellent overall stereochemical parameters. Furthermore, 97.7% of residues were located within the most favored regions of the Ramachandran plot, with no residues in disallowed regions. The model also exhibited no steric clashes or bad contacts, indicating accurate backbone geometry and proper residue packing.

Structural quality assessment of the StructiRBD-HA-VX vaccine construct identified model 2.2 as the most suitable candidate for docking analysis among the refined models. This selection was supported by several key quality indicators: a high percentage of residues in the Ramachandran favored regions (96.7%), no residues in disallowed regions, minimal bond length and angle deviations (5.1), absence of steric clashes (bad contacts), and a strong overall G-factor (0.21). Collectively, these metrics indicate that model 2.2 possesses excellent stereochemical quality and backbone geometry.

Structural quality assessment of the refined FusiCon-HA-VX vaccine constructs using PROCHECK identified model 2.2 as the most suitable candidate for further analysis, including protein–protein docking. This model exhibited the highest overall G-factor (0.29), indicating superior stereochemical quality across dihedral angles, bond lengths, and bond angles. Additionally, it showed no steric clashes (bad contacts), which reflects optimal stereochemical features. While its Ramachandran plot showed a slightly lower core region occupancy (97.4%) compared to other models, this value remains well above the acceptable threshold (>90%), confirming excellent backbone geometry. The model also demonstrated minimal bond length and angle deviation (4.6), further supporting its high-quality structural characteristics. These combined metrics establish model 2.2 as the best candidate for subsequent docking studies.

Structural quality assessment of the 400aa HA fragment identified model 2 as the most suitable candidate for further analysis. The model showed a high proportion of residues in the Ramachandran favored regions (92.6%), with only 0.3% in disallowed regions, confirming reliable backbone geometry. It also demonstrated minimal bond length and angle deviations (5.8), a very limited number of steric clashes (1 bad contact), and a positive overall G-factor (0.11), supporting its stereochemical quality. Collectively, these indicators establish model 2 as the best candidate for subsequent docking studies.

### 3.8. Molecular Docking Analysis

#### 3.8.1. TLR2 and TLR4 Complexes with the EpitoCore-HA-VX Vaccine

**Figure 7:**
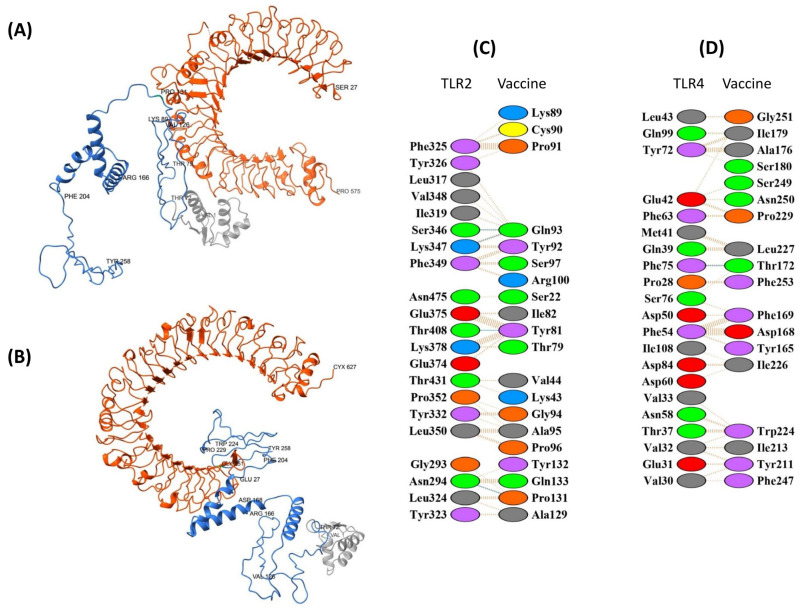
Molecular docking of the vaccine construct (blue and grey) with immune receptors (orange). (**A**) The cartoon depiction of the EpitoCore-HA-VX Vaccine-TLR2 and (**B**) TLR4 complex are illustrated using Chimera software. Panels (**C**,**D**) present detailed residue–residue interaction maps between the vaccine and TLR2 and TLR4. Interaction colors: red = salt bridges, yellow = disulphide bonds, blue = hydrogen bonds, orange dashed = non-bonded contacts. Residue colors: blue = positive (H, K, R); red = negative (D, E); green = polar (S, T, N, Q); grey = aliphatic (A, V, L, I, M); magenta = aromatic (F, Y, W); brown = Pro & Gly; yellow = cysteine.

For TLR2, the top-ranked pyDockWEB model (5647) showed a favorable total energy of −43.958 (electrostatics −32.350; van der Waals −13.955; desolvation 23.463). PDBsum indicated an interface of ~1062–1110 Å^2^ involving 21 and 20 residues on each chain, supported by four hydrogen bonds and 173 non-bonded contacts. The interaction included residues Thr79, Tyr81, Ile82, Cys90–Ser97, and Ala129–Gln133, overlapping with three predicted B-cell epitopes (Figure 7A,C).

For TLR4, model 5945 ranked highest, with a total energy of −76.752 (electrostatics −69.745; van der Waals −8.566; desolvation 15.602). The interface covered ~1088–1093 Å^2^ with 21 and 18 residues, one hydrogen bond, and 184 non-bonded contacts. Binding involved residues Asp168, Phe169, Thr172, Ile179–Ser180 (two CTL epitopes), and Tyr211–Gly251 across three HTL epitopes (Figure 7B,D).

Together, these docking results suggest plausible TLR2/4 interfaces for EpitoCore-HA-VX, highlighting potential overlap with predicted immunogenic regions. Nonetheless, these are computational predictions and should be interpreted cautiously with respect to functional receptor activation.

#### 3.8.2. TLR2 and TLR4 Complexes with the StructiRBD-HA-VX Vaccine

**Figure 8:**
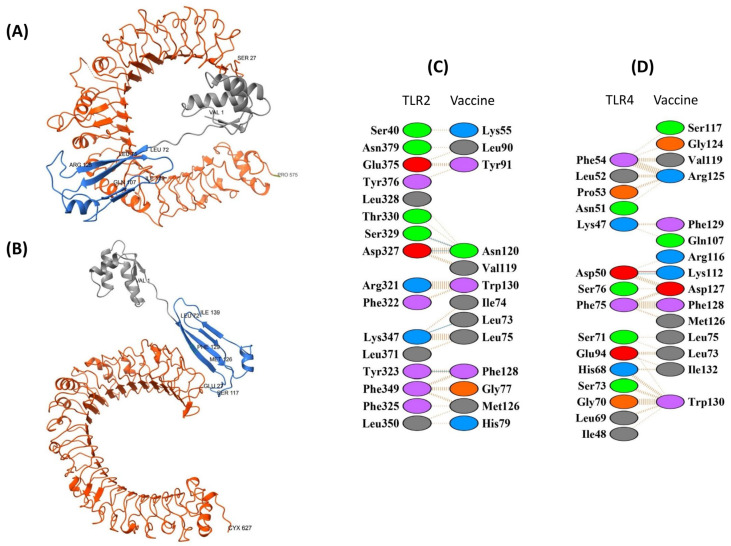
Molecular docking of the vaccine construct (blue and grey) with immune receptors (orange). (**A**) The cartoon depiction of the StructiRBD-HA-VX vaccine-TLR2 and (**B**) TLR4 complex are illustrated using Chimera software. Panels (**C**,**D**) present detailed residue–residue interaction maps between the vaccine and TLR2 and TLR4. Interaction colors: red = salt bridges, yellow = disulphide bonds, blue = hydrogen bonds, orange dashed= non-bonded contacts. Residue colors: blue = positive (H, K, R); red = negative (D, E); green = polar (S, T, N, Q); grey = aliphatic (A, V, L, I, M); magenta = aromatic (F, Y, W); brown = Pro & Gly; yellow = cysteine.

For TLR2, the best-ranked pyDockWEB model (7194) had a total energy of −41.180 (electrostatics −13.167; desolvation −29.101; van der Waals 10.881). PDBsum analysis showed an interface of ~952 Å^2^ involving 16 and 13 residues, stabilized by four hydrogen bonds and 142 non-bonded contacts. Key interacting residues included Leu73, Gln107, Lys112, Arg125, and the C-terminal segment Phe128–Lys134, overlapping with the RBD domain (Figure 8A,C). These features suggest a plausible interface consistent with epitope-rich regions.

For TLR4, two models were considered, with 6150 selected as the most balanced candidate (total energy −44.518; electrostatics −17.614; van der Waals 55.490; desolvation −32.453). The interface spanned ~684 Å^2^ with 15 residues, supported by one salt bridge, one hydrogen bond, and 172 non-bonded contacts. Interacting residues included Leu73, Leu75, Gln107, Lys112, Arg116, Ser117, Val119, and the segment Gly124–Phe129 (Figure 8B,D).

Overall, the docking predictions suggest potential contacts of StructiRBD-HA-VX with TLR2 and TLR4 through regions containing both CTL- and HTL-related residues, though these remain computational and should be interpreted cautiously.

#### 3.8.3. TLR2 and TLR4 Complexes with the FusiCon-HA-VX Vaccine

**Figure 9:**
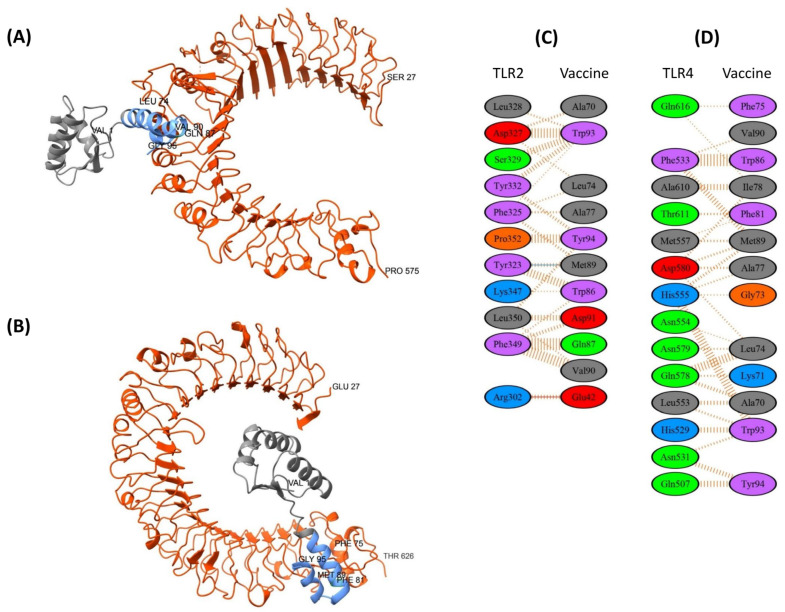
Molecular docking of the vaccine construct (blue and grey) with immune receptors (orange). (**A**) The cartoon depiction of the FusiCon-HA-VX vaccine vaccine-TLR2 and (**B**) TLR4 complex are illustrated using Chimera software. Panels (**C**,**D**) present detailed residue–residue interaction maps between the vaccine and TLR2 and TLR4. Interaction colors: red = salt bridges, yellow = disulphide bonds, blue = hydrogen bonds, orange dashed = non-bonded contacts. Residue colors: blue = positive (H, K, R); red = negative (D, E); green = polar (S, T, N, Q); grey = aliphatic (A, V, L, I, M); magenta = aromatic (F, Y, W); brown = Pro & Gly; yellow = cysteine.

For TLR2, model 5546 was considered the most plausible docking candidate, with a total energy of −46.485 (electrostatics −10.800; desolvation 64.047). PDBsum analysis indicated an interface of ~772 Å^2^, including one salt bridge, one hydrogen bond, and 165 non-bonded contacts. Interacting residues on the HA region included Leu74, Ala77, Trp86, Gln87, Met89, Val90, Asp91, Trp93, and Tyr94 (Figure 9A,C).

For TLR4, model 8441 was selected as the most balanced solution (desolvation −35.7; electrostatics −11.4; van der Waals 27.3). The interface covered ~702–778 Å^2^, involving 14 and 13 residues, supported by 138 non-bonded contacts. Key residues included Gly73, Phe75, Leu74, Ala77, Ile78, Phe81, Trp86, Met89, Val90, Trp93, and Tyr94, suggesting a predominantly hydrophobic interaction surface (Figure 9B,D).

Overall, the docking predictions suggest that FusiCon-HA-VX can engage TLR2 and TLR4 through compact interfaces enriched in hydrophobic residues. These findings provide model-based insights into possible receptor interactions, though functional implications should be interpreted cautiously.

#### 3.8.4. TLR2 and TLR4 Complexes with the 400aa Ha Fragment

**Figure 10:**
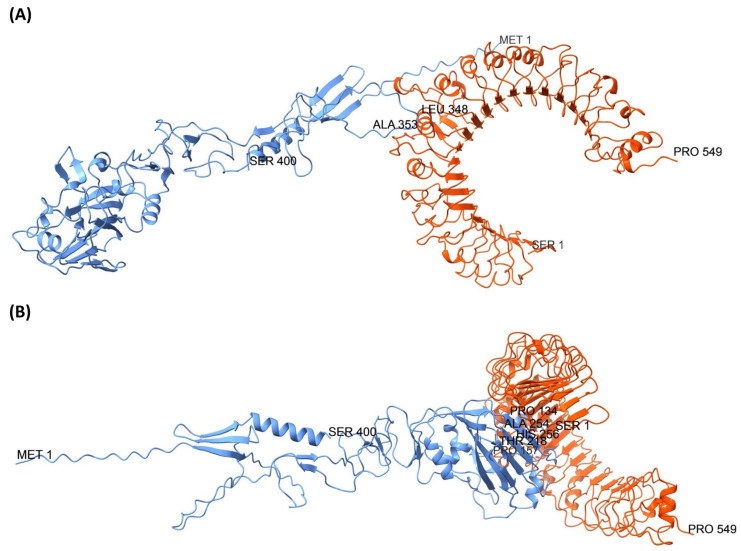
Molecular docking of the 400aa HA fragment construct (blue) with TLR2 (orange). (**A**) Docking model showing interaction of TLR2 with the fusion peptide region of HA. (**B**) Docking model highlighting binding to the RBD region of HA. Complexes were visualized using UCSF Chimera. The N- and C-terminal residues of both HA and TLR2, as well as key HA residues at the binding interfaces, are indicated.

Docking analysis of TLR2 with the 400-aa HA fragment identified two representative complexes with distinct binding sites (Figure 10). The first complex, model 7306, docked at the fusion peptide region of HA and showed a favorable binding energy (electrostatics –23,020; desolvation –18,635; van der Waals 73,173; total –34,338). PDBsum analysis revealed 34 interface residues, an interface area of 1920 Å^2^, one salt bridge, four hydrogen bonds, and 206 non-bonded contacts. Key interacting residues included Arg346, Leu348, Phe349, Gly350, Ala351, Ile352, and Ala353, supporting a stable interaction at this site. The second complex, model 1451 (ranked 5 in the pyDock scoring), targeted the RBD region of HA with a total docking energy of –25,085 (electrostatics –26,328; desolvation 2847; van der Waals –16,040). This interface involved 36 residues across 1966 Å^2^, stabilized by nine hydrogen bonds and 194 non-bonded contacts. Residues Gln131, Pro134, Ser136, Ser137, Glu142, Leu145, Lys177, Ile178, Ser179, Tyr180, and Glu267 were among those contributing to the interaction, suggesting strong intermolecular recognition despite a less favorable energetic profile compared to model 7306.

**Figure 11:**
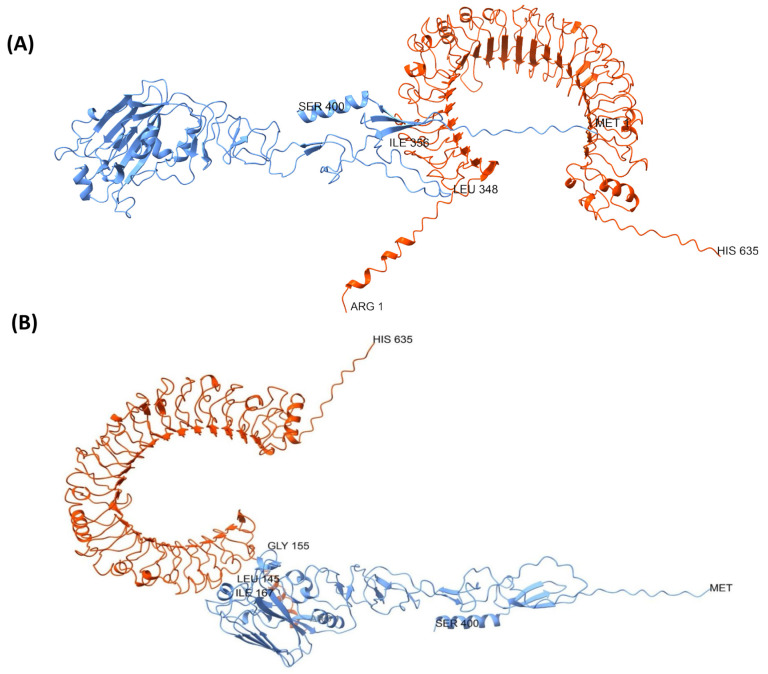
Docking analysis of the 400aa HA fragment construct (blue) with TLR4 (orange). (**A**) Model illustrating the interaction of TLR4 with the fusion peptide region of HA. (**B**) Model depicting binding at the RBD region of HA. The complexes were rendered in UCSF Chimera, with the N- and C-terminal residues of both HA and TLR4, as well as key HA interface residues, annotated.

Docking analysis of TLR4 with the 400-aa HA fragment also revealed two representative complexes involving distinct HA regions (Figure 11). Model 8984, ranked first in the pyDock scoring, bound at the fusion peptide region and exhibited the most favorable docking energy (electrostatics –16,630; desolvation –27,093; van der Waals 29,528; total –40,770). PDBsum analysis indicated 29 interface residues with an interface area of 1635 Å^2^, stabilized by two hydrogen bonds and 174 non-bonded contacts. Key interacting residues included Arg346, Leu348, Phe349, Gly350, Ala351, Ile352, Ala353, Gly354, Phe355, and Ile356, highlighting a strong and compact interaction. In contrast, model 5125 (ranked seventh) bound to the RBD region with a total docking energy of –30,679 (electrostatics –11,386; desolvation –18,578; van der Waals –7142). This interface involved 29 residues across an area of 1457 Å^2^, supported by three hydrogen bonds and 154 non-bonded contacts. Residues Leu145, Gly146, Val147, Ala149, Gly155, Ala156, Pro157, Trp165, Ile167, Lys168, Lys169, Asn170, Asp171, Asn205, and Leu206 contributed to this binding mode, suggesting a stable but comparatively weaker interaction relative to model 8984.

### 3.9. Immune Simulation

**Figure 12:**
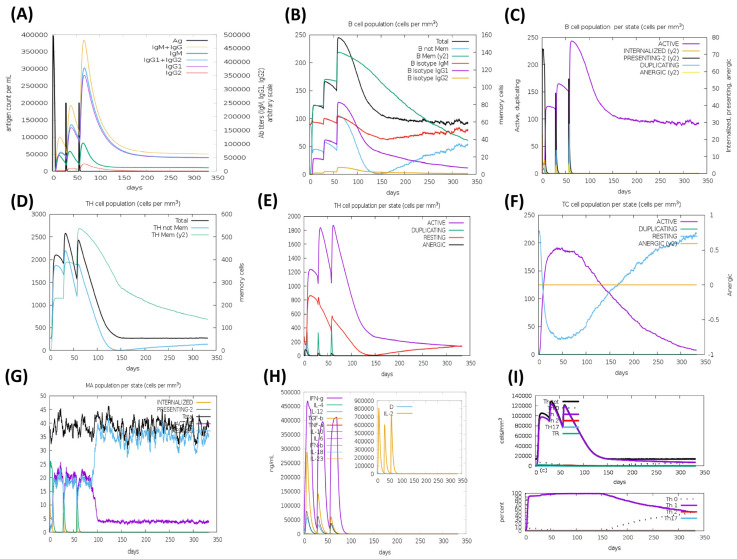
Simulated immune response to the EpitoCore-HA-VX vaccine. In silico immune response profiles generated by the C-ImmSim server for the EpitoCore-HA-VX construct are presented, illustrating the predicted dynamics of humoral and cellular immune components following vaccine administration. (**A**) The immunoglobulins and the immunocomplex response to EpitoCore-HA-VX vaccine (black vertical lines); specific subclasses are indicated as colored peaks; (**B**) B-cell populations after three injections; (**C**) Evolution of B cell; (**D**) T-helper cell populations per state after injections; (**E**) Population per state of T-helper cell; (**F**) Population per state of cytotoxic T-cell; (**G**) Population per state of macrophages; (**H**) Concentration of cytokines and interleukins. Inset plot shows danger signal together with leukocyte growth factor IL-2; (**I**) Evolution of T-helper cell classes with the course of vaccination.

**Figure 13:**
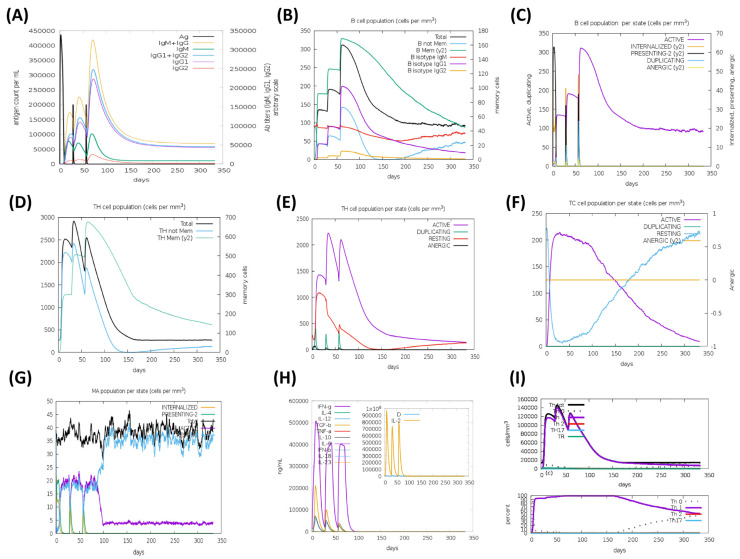
Simulated immune response to the StructiRBD-HA-VX vaccine. In silico immune response profiles generated by the C-ImmSim server for the StructiRBD-HA-VX construct are presented, illustrating the predicted dynamics of humoral and cellular immune components following vaccine administration. (**A**) The immunoglobulins and the immunocomplex response to StructiRBD-HA-VX vaccine (black vertical lines); specific subclasses are indicated as colored peaks; (**B**) B-cell populations after three injections; (**C**) Evolution of B cell; (**D**) T-helper cell populations per state after injections; (**E**) Population per state of T-helper cell; (**F**) Population per state of cytotoxic T-cell; (**G**) Population per state of macrophages; (**H**) Concentration of cytokines and interleukins. Inset plot shows danger signal together with leukocyte growth factor IL-2; (**I**) Evolution of T-helper cell classes with the course of vaccination.

**Figure 14:**
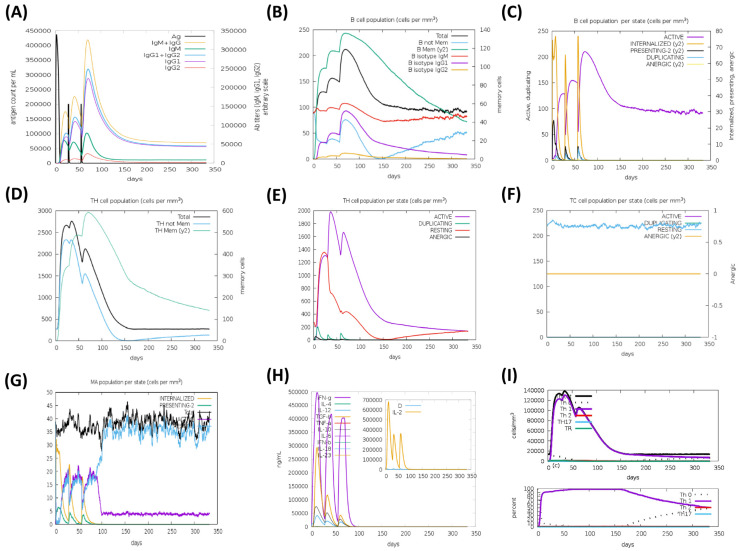
Simulated immune response to the FusiCon-HA-VX vaccine. In silico immune response pro-files generated by the C-ImmSim server for the FusiCon-HA-VX construct are presented, illustrating the predicted dynamics of humoral and cellular immune components following vaccine ad-ministration. (**A**) The immunoglobulins and the immunocomplex response to FusiCon-HA-VX vaccine (black vertical lines); specific subclasses are indicated as colored peaks; (**B**) B-cell populations after three injections; (**C**) Evolution of B cell; (**D**) T-helper cell populations per state after injections; (**E**) Population per state of T-helper cell; (**F**) Population per state of cytotoxic T-cell; (**G**) Population per state of macrophages; (**H**) Concentration of cytokines and interleukins. Inset plot shows danger signal together with leukocyte growth factor IL-2; (**I**) Evolution of T-helper cell classes with the course of vaccination.

**Figure 15:**
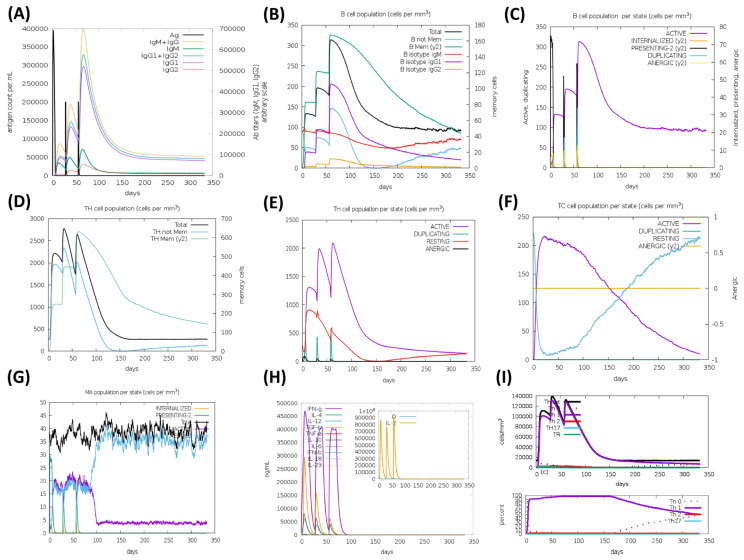
Simulated immune response to the 400aa HA fragment construct. In silico immune profiles were generated using the C-ImmSim server, illustrating the predicted dynamics of both humoral and cellular components following administration of the construct. (**A**) Immunoglobulin and immunocomplex responses to the 400aa HA fragment (black vertical lines), with specific antibody subclasses shown as colored peaks; (**B**) B-cell populations after three doses; (**C**) B-cell clonal expansion over time; (**D**) T-helper cell populations across different states after immunization; (**E**) Dynamics of T-helper cell subsets; (**F**) Dynamics of cytotoxic T-cell subsets; (**G**) Macrophage population changes during the response; (**H**) Cytokine and interleukin concentrations, with the inset highlighting danger signal and IL-2 levels; (**I**) Evolution of T-helper cell classes throughout the vaccination course.

The HA based vaccine candidates, EpitoCore-HA-VX, StructiRBD-HA-VX 2, FusiCon-HA-VX, and the 400aa Ha fragment were predicted to elicit primary and secondary immune responses upon administration, as illustrated in Figure 12, Figure 13, Figure 14 and Figure 15. The primary response was characterized by a transient elevation of IgM antibodies following the initial antigen exposure, which then declined over time. In contrast, the secondary immune response was marked by a strong production of IgG1 antibodies, along with moderate levels of IgM and IgG2. Notably, IgG1 levels remained elevated for over 300 days in the simulation outputs, suggesting the potential establishment of longer-lasting humoral responses (Figure 12A, Figure 13A, Figure 14A and Figure 15A).

This antibody profile was accompanied by an expansion of the B-cell population, particularly within the memory compartment and among B cells expressing IgM and IgG1 isotypes (Figure 12B, Figure 13B, Figure 14B and Figure 15B). State-specific analysis revealed increases in activated B cells, consistent with effective antigen recognition and ongoing immune activation (Figure 12C, Figure 13C, Figure 14C and Figure 15C).

All three vaccine constructs also promoted the expansion of Th cells. Following immunization, memory Th cell populations increased and, although they declined slightly over time, they remained detectable up to 300 days in the simulation, consistent with the potential formation of a memory pool (Figure 12D, Figure 13D, Figure 14D and Figure 15D). Activated Th cells peaked shortly after immunization and then decreased, while resting Th cells progressive increased, reflecting a modeled shift toward the establishment of memory (Figure 12E, Figure 13E, Figure 14E and Figure 15E).

A cytotoxic T cell response was also predicted, particularly for EpitoCore-HA-VX (Figure 12F) and StructiRBD-HA-VX (Figure 13F), and the 400a HA fragment (Figure 15F). These candidates showed a rise in activated TC cells shortly after immunization, followed by a gradual decline, while resting (naïve and memory) TC cells steadily increased. This pattern is consistent with a modeled transition from effector activity toward longer-term cellular memory. By contrast, the FusiCon-HA-VX vaccine did not show a notable cytotoxic T-cell response in silico (Figure 14F).

Macrophage dynamics showed increases in both active and resting populations after immunization with all constructs (Figure 12G, Figure 13G, Figure 14G and Figure 15G). Active macrophages began to decline approximately 30 days after the final dose, while resting macrophages continued to rise, suggesting a resolution phase and return toward homeostasis.

Cytokine profiling indicated elevated IFN-γ and IL-2 following vaccination (Figure 12H, Figure 13H, Figure 14H and Figure 15H), consistent with Th1-type and cytotoxic T cell responses. TGF-β increased after the first dose but declined after subsequent immunizations, suggesting an initial regulatory phase that was later downregulated in favor of effector-biased responses.

In support of this, Th1 cell counts rose after each vaccination, peaking at approximately 140,000 cells/mm^3^ following the third dose, and then declining to ~20,000 cells/mm^3^ by day 150, where they plateaued (Figure 12I, Figure 13I, Figure 14I and Figure 15I). This trajectory suggests the modeled development of a Th1 memory population capable of long-term immune surveillance.

Taken together, the simulations suggest that all three vaccine candidates could promote broad immune response, including strong humoral, helper T-cell, and innate immune activation. The sustained IgG1 levels, memory B and T cells, and Th1-associated cytokines such as IFN-γ and IL-2 are indicative of effective priming in silico, though their persistence and magnitude in vivo remain to be validated experimentally. Although FusiCon-HA-VX did not elicit a discernible cytotoxic T-cell response in the model, it nevertheless supported antibody-mediated and helper T-cell immunity, underscoring its potential as a subtype-specific vaccine candidate.

### 3.10. Population Coverage Analysis

Population coverage analysis was performed using the selected vaccine epitopes and their corresponding HLA class I and class II restriction alleles. The results suggest that the designed vaccines could provide very broad global population coverage, with combined class I and II predictions approaching 100%. This indicates that at least one epitope is likely to be presented by HLA molecules across all major world populations. When analyzed separately, the projected global coverage was 63.96% for HLA class I and 100% for HLA class II, underscoring the stronger contribution of class II epitopes to overall coverage.

Geographical distribution analysis further supports the broad potential of the vaccine designs. Most regions—including Central Africa, Central America, East Africa, East Asia, Europe, North Africa, North America, Northeast Asia, Oceania, South America, South Asia, Southeast Asia, Southwest Asia, West Africa, and the West Indies—showed full coverage in the combined analysis. The only exception was South Africa, where predicted coverage was slightly lower at 83.91%, likely reflecting regional differences in HLA allele frequencies not optimally matched by the selected epitopes.

Taken together, these findings indicate a promising breadth of immunogenic potential across diverse human populations. At the same time, the reduce coverage predicted for South Africa highlights the value of considering region-specific epitopes in future refinements for further enhance inclusivity and protective reach.

### 3.11. Codon Optimization and In Silico Cloning of Vaccine Constructs

To enhance translational efficiency and maximize protein expression in a bacterial host, all vaccine constructs were codon-optimized for expression in *E. coli* strain K-12. The optimized sequence of EpitoCore-HA-VX comprised 257 amino acids (771 bp), with a Codon Adaptation Index (CAI) [34] of 0.997 and a GC content of 49.7%, both indicative of efficient expression in *E. coli*. The StructiRBD-HA-VX construct encoded 124 amino acids (372 bp) and achieved a perfect CAI of 1.0, with GC content of 49.4%. Similarly, FusiCon-HA-VX consisted of 95 amino acids (285 bp), also with a CAI of 1.0 and a GC content of 48.4.%, supporting its suitability for high-level expression in the bacterial system. The 400-aa HA fragment likewise exhibited a CAI of 1.0 and a GC content of 49.1%, consistent with optimal codon usage for efficient bacterial expression.

To facilitate downstream cloning, *Xho*I and *BamH*I restriction sites—absent from the native vaccine sequences—were introduced at the 5′ and 3′ termini, respectively. The codon-optimized cDNA sequences were then cloned in silico into the pET28a(+) expression vector using SnapGene, allowing for precise visualization and validation of the cloning strategy.

### 3.12. Comparative Evaluation of Vaccine Constructs and HA Fragment

To contextualize the design and performance of the engineered vaccine candidates, we conducted a side-by-side comparison of EpitoCore-HA-VX, StructiRBD-HA-VX, FusiCon-HA-VX, the 400-aa HA fragment, and the previously reported HA-13–263-Fd-His construct (an external, literature-derived comparator comprising HA1 residues 13–263 fused to a T4 fibritin foldon and a 6×His tag). The two reference sequences (400-aa HA and HA-13–263-Fd-His) [35] were analyzed with the same bioinformatic pipeline as our three candidates—including epitope origin and conservation, antigenicity/safety screens, stereochemical assessment, TLR2/TLR4 docking and interface analysis, immune simulation outputs, population coverage, and codon-optimization profiles—to enable like-for-like comparison. The comparative overview highlights both the unique features of each designed construct and their relative positioning versus the reference comparators, providing a consolidated perspective on immunological potential and priorities for experimental follow-up (Table 5).

## 4. Discussion

The present study highlights the feasibility of rationally designing influenza A vaccine candidates that emphasize conserved elements of the HA glycoprotein—particularly within the RBD and adjacent functional regions—as a strategy to overcome antigenic variability. By prioritizing sequences that are both functionally essential and evolutionarily stable, this approach aims to provide protection not only against diverse H5N1 strains but also across multiple influenza A subtypes, thereby contributing to the broader pursuit of a universal influenza vaccine.

To this end, extensive HA sequence datasets were analyzed to identify conserved immunologically relevant regions. More than 20,000 H5N1 HA sequences were aligned to derive a master consensus RBD (residues 124–277), encompassing residues critical for receptor engagement. In parallel, over 190,000 HA sequences representing multiple influenza A subtypes were screened to identify a highly conserved segment spanning the HA1–HA2 junction that contains the fusion peptide, a key mediator of membrane fusion and viral entry. These analyses are consistent with prior studies showing that broadly neutralizing antibodies frequently target conserved regions of HA—including the RBD, fusion peptide, and inter-monomeric interfaces [36,37]. Findings from these datasets informed the design of three distinct vaccine constructs, each incorporating a common adjuvant sequence derived from the 50S ribosomal protein to optimize immunogenicity. Although clinical use of L7/L12 as an adjuvant is not yet established, its reported immunostimulatory activity in preclinical models and the optimized truncated variant used here support its consideration as a research adjuvant; future work will be needed to compare its reactogenicity and safety in relevant experimental systems.

The first construct, EpitoCore-HA-VX, was an H5N1-specific multi-epitope vaccine containing predicted B-cell, CTL, and HTL epitopes, without prioritizing native conformational preservation. The second, StructiRBD-HA-VX, retained the native structure of an H5N1 consensus RBD fragment (residues 188 to 255), preserving conformational epitopes likely to enhance antibody recognition. The third, FusiCon-HA-VX, targeted the highly conserved HA1–HA2 junction across more than 20 influenza A subtypes, adopting an α-helical conformation in the final construct. While the first two constructs emphasize subtype-specific protection, FusiCon-HA-VX represents a candidate with the potential for broader cross-subtype immunity. Similar universal vaccine strategies have targeted conserved RBS epitopes (e.g., CH65) [38] or employed consensus- or chimeric-based HA constructs (e.g., COBRA or headless HA) [39,40], though outcomes across subtypes have been variable [41].

To contextualize these designs, we also analyzed two HA comparators under the same bioinformatic pipeline: (i) a 400-aa HA fragment and (ii) the previously reported HA-13–263-Fd-His construct spanning HA1 residues 13–263. Both reference sequences were evaluated using identical methods (antigenicity/safety screening, structural refinement and stereochemical assessment, TLR2/TLR4 docking with interface analysis, in silico immune simulation, population coverage, and codon optimization) to enable direct, side-by-side comparison with the three vaccine candidates.

Previous studies reported that immunization of mice with a 38-residue HA2 fusion peptide conferred complete protection against both homologous H3N2 and heterologous H1N1 viral challenge. However, that peptide was derived from a single isolate, limiting breadth. In contrast, our FusiCon-HA-VX incorporates a shorter, 24-residue consensus fusion peptide derived from over 190,000 sequences spanning more than 20 subtypes. Compared to the earlier 38-residue peptide [42,43], our design includes several amino acid differences that reflect evolutionary conservation rather than isolate-specific features, with potential advantages in (i) cross-subtype breadth, (ii) manufacturability while retaining key epitopes, and (iii) reduced risk of immune escape.

All three constructs—and the two comparators (400-aa HA fragment and HA-13–263-Fd-His)—were predicted in silico to be antigenic, non-allergenic, and non-toxic, supporting their suitability for preclinical evaluation. These predictions are consistent with established immunoinformatics frameworks used to pre-assess immunogenicity and safety [44,45]. In addition, several reports have shown concordance between computational epitope selection and laboratory outcomes—for example, conserved HA/NA epitopes designed in silico that elicited neutralizing antibodies in animals, and fusion-peptide–focused immunogens that induced cross-reactive and protective responses—supporting the translational potential of a conservation-guided design pathway [40,46,47].

Population coverage analysis suggested very broad applicability, with combined class I/II HLA binding predictions approaching 100% globally. Most regions reached full coverage in the combined analysis; the exception was South Africa, where predicted coverage was modestly lower (83.91%), likely reflecting regional HLA allele distributions and highlighting opportunities for localized refinement. This observation aligns with prior epitope-based vaccine studies that incorporate global HLA coverage as a design criterion [48].

Molecular docking predicted plausible interfaces of all constructs with TLR2 and TLR4, characterized by substantial buried surface areas, hydrogen bonds, and favorable electrostatic/desolvation terms. While docking is a computational approximation and does not establish receptor activation, these models are consistent with potential engagement of innate receptors—an often-used strategy to enhance vaccine immunogenicity [49,50,51]. Distinct binding profiles among the constructs may indicate some structural adaptability across immune environments.

Immune simulations suggested that all three vaccine constructs and the two comparators could elicit broad humoral and cellular responses in silico. Each formulation was associated with sustained IgG1 production in the model, along with memory B-cell and helper T-cell populations and macrophage activation, consistent with the potential for prolonged responses. Cytotoxic T-cell responses were predicted primarily for the two H5N1-focused constructs, whereas the universal fusion-peptide construct did not show a notable CTL signal, indicating possible differences in modeled capacity to drive cellular cytotoxicity. In practice, induction of CTL responses will depend on the delivery platform: nucleic acid vaccines favor MHC-I presentation, while protein formulations require efficient cross-presentation. Our constructs are compatible with both strategies, but in vivo validation will be essential to confirm CTL priming capacity. These findings are in line with previous in silico vaccine design studies in which modeled humoral/cellular trends have guided antigen prioritization [44,45]. Importantly, the outputs of immune simulations are scenario-dependent and intended for qualitative interpretation; C-ImmSim reproduces general features of primary/secondary responses but does not yield quantitative predictions of antibody magnitude or durability in humans. Accordingly, we used these results to compare relative trends across constructs (e.g., differences in IgG1-like kinetics or cellular responses) rather than to infer absolute titers or protection. Experimental validation will be essential to confirm the magnitude and persistence of the responses suggested by these models.

Codon optimization and in silico cloning further confirmed translational feasibility, with all three constructs achieving high Codon Adaptation Index values (0.997–1.0), balanced GC content (50.73%), and successful insertion into the pET28a(+) vector for *E. coli* K-12 expression. Beyond bacterial expression—which remains attractive for rapid, cost-effective screening—alternative systems will be relevant for translational development. Mammalian (HEK293, CHO) and insect (baculovirus) platforms provide glycosylation and folding environments compatible with HA, and have been used successfully in licensed or advanced influenza subunit vaccines (e.g., Flublok^®^), while yeast (Pichia pastoris) and plant-based systems offer additional options.

Progression toward translational application will likely benefit from glycosylation-competent hosts to maintain conformational epitopes and optimize immunogenicity [52,53,54]. Consistent with this need, a key limitation of the present work is that neither glycosylation nor native trimeric organization was explicitly modeled, despite their known influence on B-cell epitope exposure. Even so, many conserved epitopes (e.g., HA stem and fusion peptide) are less affected by glycan shielding, and recombinant non-glycosylated HA proteins have elicited protective responses in animal models [55,56]. Accordingly, future work in mammalian or insect systems will compare glycosylated versus non-glycosylated forms and incorporate trimeric assemblies. More broadly, because all findings derive from computational analyses, this study should be viewed as a preclinical in silico framework whose value lies in prioritizing promising constructs and guiding subsequent wet-lab efforts. Because our epitope predictions are tailored to human HLA alleles, bridging studies in humanized HLA mice and conventional murine H-2 mapping will be needed to assess cross-reactivity. In parallel, ferret challenge models—widely used in influenza research—will provide critical efficacy data despite differences in epitope presentation. Experimental testing in appropriate animal models (including humanized HLA mice, conventional mouse/ferret challenge models, and BSL-3 studies for highly pathogenic strains) will be essential to confirm immunogenicity, safety, and protective efficacy.

This work is distinct from prior efforts in several ways. First, it directly compares three strategies—multi-epitope design (EpitoCore-HA-VX), structurally preserved RBD (StructiRBD-HA-VX), and minimal consensus fusion peptide (FusiCon-HA-VX)—within a harmonized computational framework, enabling head-to-head evaluation rather than isolated case studies [44,57,58]. Second, the study analyzed an unusually large number of sequences (>20,000 H5N1 sequences for RBD consensus and >190,000 across subtypes for the HA1–HA2 junction), giving a much clearer picture than previous work. Third, by comparing different design strategies—some that combine epitopes without keeping their structure, others that preserve structure, and others that use a universal junction—the study points to important differences that appear to have received limited side-by-side evaluation in prior work. Fourth, the pipeline integrates systems-level pre-assessment—including non-allergenicity/toxicity, global HLA coverage, innate receptor docking, immune simulations, and codon optimization—creating a data-rich foundation for experimental translation.

## 5. Conclusions

This study illustrates the feasibility of using computational vaccinology to prioritize both subtype-focused and cross-subtype influenza A candidates by targeting conserved regions within HA. Three constructs—EpitoCore-HA-VX, StructiRBD-HA-VX, and FusiCon-HA-VX—were developed and, in silico, were predicted to be antigenic while non-allergenic and non-toxic, to exhibit broad projected HLA coverage, and to form plausible TLR2/TLR4 interfaces. Immune simulations suggested the potential for robust humoral responses and, for the H5N1-focused designs, modeled cytotoxic T-cell activity; codon optimization and in silico cloning supported practical expression. To contextualize these findings, two external HA comparators—the 400-aa HA fragment and the literature-reported HA-13–263-Fd-His—were analyzed under the same pipeline, enabling side-by-side benchmarking across antigenicity/safety screens, stereochemical quality, receptor docking, immune simulations, population coverage, and codon usage.

These results are model-based and should be interpreted qualitatively: docking does not establish receptor activation, population-coverage outputs are estimates, and immune-simulation durability is predictive rather than quantitative. Glycosylation and native trimeric organization were not explicitly modeled and will be important to address in glycosylation-competent expression systems. Taken together, the integrated workflow—epitope prioritization, structural modeling/refinement, docking, and immune simulation—provides a rational, adaptable framework for ranking HA-based vaccine concepts and relevant comparators, with potential applicability to other rapidly evolving pathogens; experimental validation will be essential to confirm immunogenicity, safety, and protective efficacy.

## Figures and Tables

**Table 1:** Number of Hemagglutinin (HA) Sequences Retrieved from Various Influenza A Subtypes.

Influenza A Subtypes	Number of HA Sequences
H1N1	49,050
H1N2	7030
H3N2	56,119
H5N1	20,039
H5N2	1835
H5N3	278
H5N6	926
H5N8	1229
H6N1	798
H6N2	1045
H7N2	448
H7N3	1068
H7N4	50
H7N7	511
H7N9	1502
H9N2	13,270
H10N3	184
H10N4	105
H10N5	158
H10N7	826
H10N8	141

**Table 2:** Computationally Predicted linear B-cell Epitopes.

Rank	Sequence	Start Position *	Score
1	TNLYKNPTTYISVGTS	81	0.94
2	CPYQGAPSFFRNVVWL	28	0.94
3	SQVNGQRGRMDFFWTI	110	0.89
4	DLLILWGIHHSNNAEE	64	0.86
5	TIKISYNNTNREDLLI	52	0.86
6	IQIIPKSSWPNHETSL	7	0.84

The predicted B cell epitopes are ranked according to their score obtained by trained recurrent neural network. Higher score of the peptide means the higher probability to be as epitope. All the peptides shown here are above the threshold value chosen. * Epitope positions are numbered relative to the 124–277 HA RBD fragment used as input for prediction.

**Table 3:** CTL epitopes prediction for the input RBD of HA protein sequences.

Sequence Number	Sequence	Score	Supertype
1	RMDFFWTIL	0.9613	A2
2	YISVGTSTL	0.9443	A2
3	RLAPKIATR	1.3918	A3
4	TLNQRLAPK	1.3917	A3
5	MDFFWTILK	1.3062	A3
6	NLYKNPTTY	1.1584	A3
7	FIAPEYAYK	1.1506	A3
8	APSFFRNVV	1.6389	B7
9	CPYQGAPSF	1.4493	B7
10	APKIATRSQ	1.1018	B7
11	APEYAYKIV	1.0909	B7
12	YISVGTSTL	0.9547	B7

Threshold value: 0.75, Combined scores.

**Table 4:** HTL epitopes prediction for the input RBD of HA protein sequences.

Rank	Allele	Epitope	Method	Percentile Rank	Score	IF-g Inducer	
						Result	Score
1	HLA-DRB1 08:02	FIAPEYAYKIVKKGD	netmhciipan_el 4.1	0.11	0.8720	Positive	1
2	HLA-DRB3 01:01	WTILKPDDAIHFESN	netmhciipan_el 4.1	0.12	0.8699	Positive	0.45
3	HLA-DQA1 04:01/DQB1 04:02	FIAPEYAYKIVKKGD	netmhciipan_el 4.1	0.15	0.3705	Positive	1
4	HLA-DRB3 01:01	FWTILKPDDAIHFES	netmhciipan_el 4.1	0.15	0.8370	Positive	0.47
5	HLA-DRB3 01:01	TILKPDDAIHFESNG	netmhciipan_el 4.1	0.18	0.7780	Negative	1
6	HLA-DRB3 02:02	AIHFESNGNFIAPEY	netmhciipan_el 4.1	0.18	0.8016	Positive	0.5
7	HLA-DRB1 07:01	PTTYISVGTSTLNQR	netmhciipan_el 4.1	0.21	0.8852	Positive	0.54
8	HLA-DRB3 02:02	DAIHFESNGNFIAPE	netmhciipan_el 4.1	0.22	0.7782	Positive	0.54
9	HLA-DRB3 01:01	FFWTILKPDDAIHFE	netmhciipan_el 4.1	0.26	0.7497	Positive	0.52
10	HLA-DRB3 02:02	QTNLYKNPTTYISVG	netmhciipan_el 4.1	0.31	0.7183	Positive	0.55

**Table 5:** Comparative Features of the Designed HA-RBD Vaccine Constructs and External HA Comparators (400-aa HA Fragment and HA-13–263-Fd-His).

Feature	EpitoCore-HA-VX	StructiRBD-HA-VX	FusiCon-HA-VX	HA	HA-13–263-Fd-His
Epitope Source	Multi-epitope collation (HA-RBD)	Structure-preserved RBD fragment (188–255)	Fusion peptide (HA2, 24 aa)	HA fragment (400 aa)	RBD (HA1, H5N1; 13–263)
Conservation	Consensus from >20 k H5N1	Same	Ultra-conserved across influenza A subtypes	Consensus from >20 k H5N1	Strain-specific (A/Anhui/1/2005)
Antigenicity & Safety	Antigenic; non-allergen; non-toxic	Antigenic; non-allergen	Antigenic; non-allergen	Antigenic; non-allergen	Antigenic; non-allergen
Structural Quality Assessment	Stable after refinement (97.7% favored residues, G-factor 0.21)	High stereochemical quality (96.7% favored, no clashes)	Excellent stereochemistry (97.4% favored, G-factor 0.29)	Model 2 selected; 92.6% favored residues, 0.3% disallowed; minimal deviations (5.8), 1 clash, G-factor 0.11	Good stereochemistry (91.7% favored; 0.8% disallowed; no clashes; G-factor 0.09)
Docking—TLR2	Strong (–43.9); 1062 Å^2^; engages B-cell epitopes	Strong (–41.1); 952 Å^2^; engages RBD motifs	Strong (–46.5); 772 Å^2^; engages FP residues	Two modes: FP site (–34,338; 1920 Å^2^; 34 residues; 4 H-bonds, 206 contacts); RBD site (–25,085; 1966 Å^2^; 36 residues; 9 H-bonds, 194 contacts)	Strong (–29.33); 1635 Å^2^; three binding residues in RBD domain
Docking—TLR4	Very strong (–76.7); 1088 Å^2^; engages CTL + HTL epitopes	Balanced (–44.5); 684 Å^2^; engages RBD residues	Good (702–778 Å^2^; 138 contacts, hydrophobic)	Two modes: FP site (–40,770; 1635 Å^2^; 29 residues; 2 H-bonds, 174 contacts); RBD site (–30,679; 1457 Å^2^; 29 residues; 3 H-bonds, 154 contacts)	Strong (–30.1); 1874 Å^2^; predominantly RBD-mediated
Immune Simulation	IgG1 durable >300 d; CTL + Th1 memory strong; IFN-γ/IL-2 high	Similar profile; strong CTL + Th1; durable memory	IgG1 durable >300 d; strong Th1; no CTL induction	Similar to EpitoCore/StructiRBD; in some antibody responses slightly higher	Similar to EpitoCore/StructiRBD; in some antibody responses slightly higher
Population Coverage	100% combined (class I ~64%, class II 100%)	Same	Same	Similar to EpitoCore/StructiRBD (broad global coverage)	Same
Codon Optimization	CAI 0.997; GC 49.7%; cloned in pET28a(+)	CAI 1.0; GC 49.4%	CAI 1.0; GC 48.4%	CAI 1.0; GC 49.1%	CAI 1.0; GC 50.3%

## Data Availability

All data used can be found in the text and tables.
